# Oxidative Stress in Diabetic Retinopathy: Pathogenic Mechanisms, Biomarkers and Clinical Implications

**DOI:** 10.3390/antiox15040425

**Published:** 2026-03-27

**Authors:** Caterina Toma, Diego Ferdeghini, Mohammad Mostafa Ola Pour, Sakthipriyan Venkatesan, Stefano De Cillà, Elena Grossini

**Affiliations:** 1 Eye Clinic, University Hospital Maggiore Della Carità, 28100 Novara, Italy; caterina.toma@maggioreosp.novara.it (C.T.); 20063448@studenti.uniupo.it (D.F.); stefano.decilla@med.uniupo.it (S.D.C.); 2 Laboratory of Physiology, Department of Translational Medicine, Università del Piemonte Orientale, Via Solaroli 17, 28100 Novara, Italy; 20046522@studenti.uniupo.it (M.M.O.P.); sakthipriyan.venkatesan@uniupo.it (S.V.); 3 Department of Health Sciences, Università del Piemonte Orientale “Amedeo Avogadro”, 28100 Novara, Italy

**Keywords:** diabetes, diabetic retinopathy, oxidative stress, mitochondria, neurovascular damage, redox biomarkers

## Abstract

Diabetic retinopathy (DR) is a leading cause of vision loss worldwide and represents a complex neurovascular complication of diabetes mellitus driven by chronic hyperglycemia. Increasing evidence identifies oxidative stress—defined as an imbalance between reactive oxygen species (ROS) production and antioxidant defenses—as a central pathogenic mechanism linking metabolic dysregulation to retinal injury. The retina is particularly vulnerable to oxidative damage due to its high metabolic demand, elevated oxygen consumption, and abundance of polyunsaturated fatty acids. Hyperglycemia activates multiple interconnected biochemical pathways, including the polyol and hexosamine pathways, protein kinase C signaling, advanced glycation end-product formation, and lipid peroxidation, all of which converge on excessive ROS production and mitochondrial dysfunction. Growing attention has focused on oxidative stress biomarkers as tools to characterize DR severity and progression. Elevated systemic markers of lipid, protein, and DNA oxidation, together with impaired antioxidant capacity, correlate with disease stage, while oxidative biomarkers detected in aqueous and vitreous humor reflect localized retinal injury. Importantly, oxidative stress biomarkers are also associated with functional outcomes, including best-corrected visual acuity and diabetic macular edema. Integration of systemic and ocular oxidative biomarkers with clinical staging may improve risk stratification and support personalized therapeutic strategies in DR.

## 1. Introduction

Diabetic retinopathy (DR) is a multifactorial microvascular complication of diabetes mellitus and a leading cause of preventable blindness worldwide. Despite major advances in metabolic control and ocular therapies, the prevalence of DR continues to increase in parallel with the global rise in diabetes, underscoring the need for a deeper understanding of its pathogenic mechanisms. Traditionally regarded as a purely vascular disorder, DR is now recognized as a complex neurovascular disease involving early neuronal dysfunction, glial activation, microvascular impairment, and chronic inflammation. These interconnected alterations progressively compromise retinal structure and function, ultimately leading to vision loss. Persistent hyperglycemia initiates a network of metabolic abnormalities that converge on oxidative stress, positioning redox imbalance as a central mechanism linking metabolic dysregulation to retinal injury [1,2,3,4,5,6,7,8,9,10,11].

Oxidative stress represents a fundamental biological process arising from an imbalance between the generation of reactive oxygen species (ROS) and the capacity of antioxidant defense systems to neutralize them. While controlled ROS production plays an essential role in normal cellular signaling and homeostasis, excessive accumulation of these reactive molecules leads to oxidative damage of lipids, proteins, and nucleic acids, ultimately impairing tissue structure and function [1,2,4,12,13]. The retina is particularly vulnerable to oxidative stress due to its high metabolic activity, abundant mitochondrial content, elevated oxygen consumption and high levels of polyunsaturated fatty acids (PUFAs), which are highly susceptible to lipid peroxidation [14,15].

In the context of diabetes, chronic hyperglycemia acts as the primary trigger for sustained oxidative stress within the retina. Elevated intracellular glucose levels promote excessive ROS generation through multiple converging metabolic pathways, including increased mitochondrial superoxide production, activation of the polyol and hexosamine pathways, enhanced formation of advanced glycation end products (AGEs), and activation of protein kinase C (PKC) signaling. Together, these mechanisms disrupt redox homeostasis and initiate a cascade of pathological events that progressively damage retinal microvascular, neuronal, and glial cells [2,3,4,5,6,12,16,17,18,19].

ROS comprise a heterogeneous group of chemically reactive molecules, including superoxide anion, hydrogen peroxide, and hydroxyl radicals, each characterized by distinct sources, reactivity, and biological effects. Among these, superoxide is widely considered the initiating ROS in diabetic tissues, giving rise to secondary reactive species that amplify oxidative and nitrosative stress. Experimental studies in vitro and in diabetic animal models consistently demonstrate increased ROS production in diabetic retinas, confirming that oxidative stress is an early and persistent feature of DR rather than a mere byproduct of late-stage disease [20,21,22,23].

Oxidative stress is increasingly recognized as a unifying mechanism underlying both non-proliferative and proliferative stages of the disease, as well as its most vision-threatening complication, diabetic macular edema (DME). Despite advances in glycemic control and the advent of anti-vascular endothelial growth factor (VEGF) therapies, current treatments primarily target downstream manifestations, while the upstream oxidative mechanisms remain largely unaddressed [5,24].

Importantly, oxidative stress does not operate in isolation but instead serves as a critical nexus linking metabolic dysregulation to inflammation, vascular dysfunction, neurodegeneration, and angiogenesis. A comprehensive understanding of the sources, targets, and consequences of oxidative stress in the diabetic retina is therefore essential for the development of more effective and disease-modifying therapeutic strategies. By elucidating how redox imbalance intersects with and amplifies diverse pathogenic pathways at molecular and cellular levels, oxidative stress emerges as both a key driver of DR pathogenesis and a promising target for early intervention and personalized therapy [12,25,26].

In this review, we summarize current evidence on the sources and consequences of oxidative stress in DR, with particular focus on oxidative stress biomarkers and their relationship with disease severity and visual outcomes.

## 2. Oxidative Stress in the Diabetic Retina: Pathophysiological Framework

Despite extensive experimental and clinical investigation, the precise molecular mechanisms through which chronic hyperglycemia leads to retinal pathology in DR remain incompletely defined. It is now widely accepted that hyperglycemia induces retinal damage through the dysregulation of multiple interconnected metabolic pathways rather than through a single linear mechanism. Central to this process is the sustained overproduction of ROS and the resulting disruption of cellular redox homeostasis, which serve as early amplifiers of hyperglycemia-induced retinal injury [2,13,18].

Classically, five major metabolic and biochemical abnormalities have been recognized as the core drivers of oxidative damage in the diabetic retina: activation of the PKC pathway, increased glucose flux through the polyol pathway, activation of the hexosamine pathway, enhanced intracellular formation of AGEs, and lipid peroxidation of PUFAs. Excessive ROS directly damage lipids, proteins, and nucleic acids and also act as signaling molecules that activate redox-sensitive transcription factors such as nuclear factor kappa-light-chain-enhancer of activated B cells (NF-κB). Through these mechanisms, oxidative stress amplifies inflammatory signaling, promotes endothelial dysfunction, and accelerates neuronal and glial injury, thereby contributing to both early and late pathological features of DR [12,16,19,20,27,28,29,30].

Major sources of ROS in DR are mitochondria and nicotinamide adenine dinucleotide phosphate (NADPH) oxidases. Mitochondria are widely recognized as the predominant source of ROS in the diabetic retina. Hyperglycemia-driven excess electron flux through the mitochondrial respiratory chain leads to electron leakage and superoxide overproduction, as demonstrated in in vitro studies using high-glucose-exposed retinal cells and in diabetic animal models [16,18,31,32,33,34,35].

In addition to mitochondria, NADPH oxidases (NOX enzymes) represent a regulated and cell-specific source of ROS in DR. Increased expression and activation of NOX isoforms have been observed in diabetic retinal vessels, inflammatory cells, and glial cells. NOX-derived ROS play a key role in endothelial dysfunction, leukocyte adhesion, and breakdown of the blood–retinal barrier [20,31,32,33,34,35,36].

Retinal endothelial cells and pericytes are primary targets of oxidative damage. Excessive ROS impair endothelial nitric oxide (NO) signaling, reduce vasodilatory capacity, and increase vascular permeability. Oxidative stress-induced pericyte apoptosis represents a critical early event leading to capillary destabilization and microaneurysm formation [37,38,39,40,41,42,43].

Increasing evidence indicates that oxidative stress also affects retinal neurons and glial cells early in the disease course. Neuronal oxidative damage contributes to synaptic dysfunction, impaired visual processing, and neurodegeneration. Müller glia and microglia respond to oxidative stress by adopting a pro-inflammatory phenotype, further amplifying retinal inflammation and ROS production [24,25,44].

The following sections outline the principal mechanisms by which oxidative stress drives retinal damage in DR.

### 2.1. Lipid Peroxidation as a Foundational Mechanism of Oxidative Retinal Injury

One of the earliest and most pervasive consequences of oxidative stress in DR is lipid peroxidation. Key retinal PUFAs, including docosahexaenoic acid (DHA), arachidonic acid, and oleic acid, are essential for photoreceptor membrane integrity and visual transduction, yet they also represent preferential targets for ROS-mediated damage. ROS attack PUFAs, initiating chain reactions that generate lipid peroxidation products, which further propagate radical formation through autocatalytic processes [13,15,24,29,45].

This process progressively disrupts membrane integrity, alters membrane fluidity, and compromises ion channel and receptor function. In the retina, the accumulation of lipid peroxidation has been strongly associated with disease duration and severity, and its products have been shown to activate pro-apoptotic pathways, inflammatory transcription factors, and angiogenic signaling cascades [24,29,45,46].

### 2.2. Polyol Pathway Hyperactivation and Redox Imbalance

The polyol pathway represents one of the most thoroughly characterized metabolic mechanisms linking hyperglycemia to oxidative stress in DR. Under normoglycemic conditions, only a small fraction of intracellular glucose enters this pathway. In hyperglycemia, aldose reductase becomes overactivated and reduces glucose to sorbitol using NADPH as a cofactor. Sorbitol is further oxidized to fructose, altering the alteration in the NADH/NAD^+^ ratio and disruption of cellular redox homeostasis.

This pathway contributes to oxidative stress through multiple mechanisms. First, sorbitol accumulation induces osmotic stress, cellular swelling, and capillary damage, as sorbitol cannot freely diffuse across cell membranes. Second, excessive NADPH consumption limits glutathione (GSH) regeneration, weakening antioxidant capacity. Third, the altered NADH/NAD^+^ ratio disrupts cellular redox balance and promotes ROS generation via NADH-dependent oxidases [28,45,47,48,49,50].

### 2.3. AGEs Formation and Receptor for AGE-Dependent Oxidative Amplification

AGEs arise from non-enzymatic reactions between reducing sugars or their degradation products and amino groups on proteins, lipids, and nucleic acids. While glucose itself is relatively weakly reactive, highly reactive dicarbonyl compounds—such as methylglyoxal, glyoxal, and 3-deoxyglucosone—are abundantly generated under hyperglycemic and oxidative conditions and act as potent glycating agents.

AGEs exert deleterious effects both structurally and through receptor-mediated signaling. Structurally, AGEs cross-linking stiffens extracellular matrix components, stiffen capillary basement membranes, and impairs diffusion of growth factors and nutrients. Experimental studies in cell culture systems and diabetic animal models have shown that AGEs bind to the receptor for AGEs (RAGE), triggering activation of redox-sensitive signaling pathways including NF-κB. RAGE activation stimulates NADPH oxidase and mitochondrial ROS production, promotes inflammatory cytokine release, enhances VEGF expression, induces pericyte apoptosis, and disrupts endothelial barrier function. Importantly, oxidative stress itself accelerates AGEs formation and upregulates RAGE expression, establishing a self-perpetuating cycle that sustains retinal injury. While these mechanisms have been primarily characterized in experimental models, increased AGEs levels have also been documented in patients with DR, supporting their potential clinical relevance [28,38,41,45,51,52,53,54,55,56].

### 2.4. PKC Pathway Overactivation and Oxidative Signaling Crosstalk

PKC signaling is another classical pathway activated by hyperglycemia-induced oxidative stress. Elevated intracellular glucose increases diacylglycerol (DAG) synthesis, a key activator of PKC isoforms, particularly PKC-β. PKC activation induces a wide range of pathogenic effects, including reduced NO bioavailability, increased endothelin-1 production, enhanced thromboxane synthesis, VEGF upregulation, and phosphorylation of tight junction proteins leading to increased vascular permeability. PKC also promotes ROS generation by facilitating assembly and activation of NADPH oxidase complexes, thereby contributing to sustained redox imbalance [27,45,57,58].

### 2.5. Hexosamine Pathway Flux and O-GlcNAc-Mediated Dysfunction

Increased flux through the hexosamine biosynthetic pathway represents an additional mechanism by which oxidative stress mediates retinal damage. Hyperglycemia increases levels of UDP-N-acetylglucosamine, which serves as a substrate for O-GlcNAcylation of transcription factors, enzymes, and structural proteins. Excessive O-GlcNAcylation impairs mitochondrial and endothelial function, increases vascular permeability and promotes angiogenesis [24,59,60,61].

### 2.6. Mitochondrial Superoxide Production as a Unifying Mechanism

Increasing evidence supports the concept that these distinct pathogenic pathways converge on a common upstream driver—mitochondrial superoxide overproduction within the electron transport chain [30]. Under chronic hyperglycemic conditions, increased glucose oxidation enhances electron flux through the mitochondrial respiratory chain, resulting in excessive superoxide generation. This mitochondrial-derived ROS acts as an upstream trigger coordinating activation of multiple downstream damaging pathways, including the polyol pathway, AGEs product formation, PKC signaling, and hexosamine pathway flux.

Mitochondrial superoxide overproduction promotes these processes primarily through inhibition of the glycolytic enzyme glyceraldehyde-3-phosphate dehydrogenase (GAPDH) and subsequent accumulation of upstream glycolytic intermediates which are redirected into alternative pathogenic pathways. By inducing persistent molecular alterations, this mechanism sustains oxidative stress and pathogenic signaling even after restoration of normoglycemia. This provides a mechanistic basis for the phenomenon of metabolic memory.

In addition, mitochondrial superoxide overproduction promotes secondary activation of other reactive oxygen species-generating systems, including NADPH oxidases and uncoupled endothelial NO synthase, thereby amplifying oxidative and nitrosative stress [6,18,30,62,63,64,65].

### 2.7. Mitochondrial Dysfunction and DR

As previously discussed, mitochondrial dysfunction occupies a central position in oxidative stress–mediated DR pathogenesis. In this context, chronic hyperglycemia acts as a metabolic disruptor that triggers a cascade of mitochondrial alterations.

Evidence from in vitro studies and diabetic animal models indicates that, under diabetic conditions, excessive glucose metabolism stimulates mitochondrial ROS overproduction. These ROS induce lipid peroxidation, protein modification, and mitochondrial DNA (mtDNA) damage. In experimental settings, these genetic alterations impair oxidative phosphorylation, resulting in reduced ATP production and loss of mitochondrial membrane potential. The resulting bioenergetic failure and calcium dysregulation promote opening of the mitochondrial permeability transition pore and activation of apoptotic pathways [45,61,66,67].

It is to note that mitochondria may play a role in DR through mitophagy (mitochondrial autophagy), as well. Mitophagy is a selective quality-control mechanism that eliminates damaged or dysfunctional mitochondria. In DR, its role is bidirectional and duration-dependent. In early/mild hyperglycemia, mitophagy may increase as a protective stress response to clear damaged organelles and maintain cellular viability. In persistent or severe hyperglycemia, sustained high glucose may suppress mitophagy leading to the accumulation of dysfunctional mitochondria [61].

Other mechanisms through which mitochondria contribute to the pathophysiology of DR include mitochondrial dynamics (fusion and fission) and the maintenance of the blood–retinal barrier (BRB). Maintaining a healthy mitochondrial network requires a balance between fusion (merging) and fission (splitting) [68]. In diabetic environments an imbalance favoring excessive fission, driven by the upregulation of proteins like dynamin-related protein 1, could arise. This would result in mitochondrial fragmentation, which facilitates cytochrome c release and apoptosis in retinal vascular endothelial cells and pericytes. Furthermore, mitochondrial dysfunction is a core driver of BRB destruction. The loss of ATP synthesis and oxidative stress in constituent cells—including retinal endothelial cells, retinal pigment epithelial cells, and pericytes—could lead to the breakdown of tight junctions and increased vascular permeability [69].

For all the above reasons, restoring mitochondrial plasticity and function has increasingly been considered a primary target for developing innovative DR therapies.

### 2.8. Oxidative Stress-Induced Apoptosis and Inflammation

Apoptosis of retinal endothelial cells, pericytes, neurons, and glia is a defining feature of DR and occurs early in disease progression. Oxidative stress increases mitochondrial permeability, triggering the release of pro-apoptotic proteins and ultimately leading to DNA fragmentation and cell death. NF-κB, previously described as a redox-sensitive transcription factor, also regulates inflammation and apoptosis in this context. ROS-mediated NF-κB activation induces cytokine expression and upregulation of inducible NO synthase, leading to increased NO production. NO reacts with superoxide to form peroxynitrite, a potent oxidant capable of nitrating proteins and damaging DNA. The combined effects of apoptosis and inflammation contribute to pericyte dropout, capillary degeneration, breakdown of the BRB, and DME [29,70,71,72].

### 2.9. Nitrosative Stress and Reactive Nitrogen Species

Nitrosative stress, characterized by excessive production of reactive nitrogen species (RNS) such as peroxynitrite, represents an additional dimension of redox imbalance in DR. Increased levels of nitrotyrosine indicate protein nitration and impaired NO signaling. Nitrosative stress contributes to endothelial dysfunction, neuronal injury, and breakdown of the blood–retinal barrier, further amplifying disease progression [13,24,71].

### 2.10. Epigenetic Reprogramming and Non-Coding RNA Regulation

Beyond acute biochemical damage, oxidative stress induces long-lasting epigenetic changes that perpetuate DR progression. Redox-sensitive enzymes regulate DNA methylation, histone modifications, and chromatin remodeling, leading to persistent repression of antioxidant and sustained activation of pro-inflammatory pathways.

Non-coding RNAs, including microRNAs and long non-coding RNAs, further modulate oxidative stress responses by targeting key regulators such as VEGF and NF-κB. Dysregulation of these RNA species amplifies mitochondrial dysfunction, angiogenesis, inflammation, and neurodegeneration, contributing to disease persistence and metabolic memory [4,16,24,45].

### 2.11. Role of Extracellular Vesicles (EVs) in DR

EVs serve as crucial mediators of intercellular communication in the diabetic eye by transporting proteins, lipids, and nucleic acids. In the context of DR, these vesicles play a dual role as both drivers of pathological progression and potential platforms for diagnosis and therapy. In a diabetic environment, chronic hyperglycemia and oxidative stress alter the cargo and secretion rate of EVs, turning them into agents of damage [73,74]. Experimental studies have shown that stressed retinal pigment epithelial cells release an increased volume of EVs loaded with VEGF and VEGF receptor mRNA, which induce tube formation in endothelial cells and drive pathological angiogenesis. Also, circulating EVs derived from platelets under hyperglycemic conditions may induce retinal endothelial injury by upregulating inflammatory pathways like toll-like receptor 4. In parallel, analyses of circulating EVs in patients with diabetes have demonstrated that IgG-loaded EVs in the plasma of diabetic patients can activate the complement system, exacerbating vascular damage. It is also noteworthy that diabetes may disrupt the normal exchange of signals between cells of the neurovascular unit. In cell-based models, pericytes under high-glucose stress transfer the circular RNA cPWWP2A to endothelial cells via EVs, which aggravates microvascular dysfunction [75].

### 2.12. Integrated Model of Oxidative Damage in Diabetic Retinopathy

**Figure 1:**
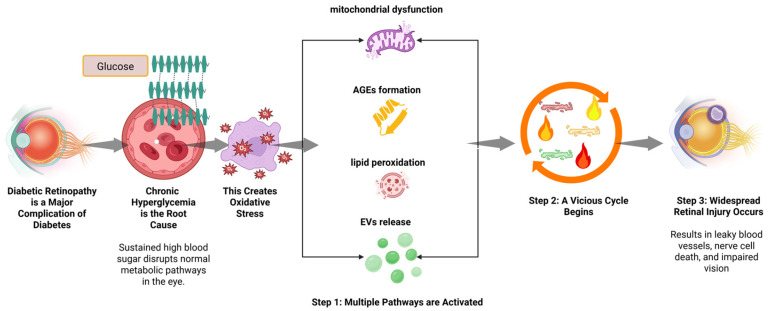
Schematic of oxidative stress-mediated pathogenesis in diabetic retinopathy. The diagram illustrates the progression from chronic hyperglycemia to retinal structural damage. Key pathways highlighted include the generation of reactive oxygen species (ROS), mitochondrial dysfunction, accumulation of advanced glycation end-products (AGEs), lipid peroxidation, and the release of pathological extracellular vesicles (EVs). These mechanisms drive a self-perpetuating cycle of inflammation and redox imbalance, leading to blood–retinal barrier breakdown, neurodegeneration, and subsequent vision loss.

Collectively, oxidative stress integrates metabolic dysregulation, mitochondrial damage, lipid peroxidation, apoptosis, inflammation, epigenetic remodeling and neurovascular dysfunction in DR. The extensive crosstalk and feed-forward amplification among these pathways explain the progressive, self-sustaining nature of the disease and underscore the need for early, multi-targeted therapeutic strategies. Experimental models of diabetes consistently demonstrate increased retinal oxidative stress, mitochondrial dysfunction, and reduced antioxidant capacity. Genetic or pharmacological interventions targeting oxidative pathways attenuate vascular leakage, neuronal loss, and inflammatory activation in preclinical studies. Clinically, elevated levels of oxidative stress biomarkers have been reported in the serum, aqueous humor, and vitreous of patients with DR. These findings correlate with disease severity, supporting the translational relevance of oxidative stress in human DR [1,24,36,38,76,77]. The complex interplay between these metabolic abnormalities and the resulting oxidative injury is summarized in Figure 1.

## 3. Biomarkers of Oxidative Stress in Diabetic Retinopathy

Oxidative stress biomarkers provide biologically meaningful evidence of redox imbalance and oxidative damage in DR. These markers reflect oxidative modifications of lipids, proteins, and nucleic acids, as well as alterations in antioxidant defense mechanisms. In DR, oxidative stress biomarkers are relevant not only for understanding disease pathophysiology but also potentially for stratifying disease severity, monitoring progression across different clinical stages, and evaluating therapeutic responses. Both systemic and ocular biomarkers have been investigated in experimental models and in human clinical studies, revealing a close relationship between systemic metabolic dysregulation, localized retinal oxidative injury, and the clinical severity of retinopathy [16,29,40].

Table 1 summarizes the main oxidative stress biomarkers used in DR.

### 3.1. Systemic Biomarkers of Oxidative Stress

#### 3.1.1. Lipid Peroxidation Markers

Markers of lipid peroxidation are among the most extensively studied systemic indicators of oxidative stress in DR. Elevated circulating levels of malondialdehyde (MDA) and 4-hydroxynonenal (4-HNE) have been consistently reported in clinical studies involving patients with diabetes, particularly in those with established retinopathy. Clinical studies have demonstrated higher circulating MDA levels in patients with non-proliferative DR (NPDR) and the highest concentrations in proliferative DR (PDR), compared with diabetic patients without retinopathy, supporting a progressive association between systemic lipid peroxidation and disease severity [40,45,78,79,80,81,82].

#### 3.1.2. Protein Oxidation and Advanced Oxidation Protein Products

Evidence from both diabetic animal models and human clinical cohorts indicates that protein oxidation represents another major consequence of sustained oxidative stress. Advanced oxidation protein products (AOPPs), carbonylated proteins, and oxidized albumin are increased in the plasma of patients with DR. These markers indicate irreversible oxidative modification of circulating proteins and are linked to activation of inflammatory pathways and endothelial dysfunction. In clinical studies, circulating AOPP levels are significantly higher in patients with NPDR and further increased in those with PDR, suggesting a stage-dependent accumulation of oxidatively modified proteins [40,45,80].

#### 3.1.3. Oxidative DNA Damage Markers

Systemic markers of oxidative DNA damage, particularly 8-hydroxy-2′-deoxyguanosine (8-OHdG), have been widely investigated in both experimental settings and human studies in DR. Elevated serum and urinary levels of 8-OHdG reflect oxidative damage to nuclear and mitochondrial DNA and have been correlated with poor glycemic control and the severity of microvascular complications, including retinopathy. Notably, higher 8-OHdG levels are more frequently observed in patients with long-standing diabetes and advanced DR, particularly PDR, indicating cumulative genomic injury. This association supports the concept that oxidative DNA damage increases progressively with disease severity and may contribute to irreversible retinal injury and metabolic memory [3,45,80,83].

#### 3.1.4. Alterations in Antioxidant Defense Systems

In parallel with increased oxidative damage markers, patients with DR often exhibit reduced systemic antioxidant capacity. Decreased levels or activity of endogenous antioxidants such as superoxide dismutase (SOD), catalase, GSH peroxidase, and reduced GSH, together with a reduction in total antioxidant status (TAS), have been reported in diabetic patients with retinopathy. Importantly, the decline in antioxidant defenses appears to be stage-dependent, with patients with non-proliferative DR showing reduced antioxidant activity and lower TAS compared with diabetic patients without retinopathy, and those with proliferative DR exhibiting the most pronounced depletion. This progressive impairment of systemic antioxidant capacity has a potential role as a biomarker of disease severity and susceptibility to ongoing oxidative damage [45,78,79,82,84].

### 3.2. Ocular Biomarkers of Oxidative Stress

#### 3.2.1. Oxidative Stress Markers in Aqueous Humor

Clinical studies analyzing aqueous humor samples from patients with DR have provided direct evidence of local oxidative stress. Elevated levels of MDA, 8-OHdG, nitric oxide (NO), and protein carbonyls have been detected in aqueous samples from patients with DR, indicating active oxidative damage within the ocular environment. In parallel, a significant reduction in total antioxidant capacity (TAC) has been consistently reported in the aqueous humor of patients with DR compared with non-diabetic controls, reflecting impaired local antioxidant defenses. These clinical findings suggest that systemic oxidative stress may be mirrored and possibly amplified locally in the eye; however, mechanistic interpretation of this relationship largely derive from experimental models. Importantly, increased aqueous humor oxidative biomarkers and reduced TAC are already detectable in patients with early and moderate NPDR, and their alterations tend to become more pronounced with disease progression. Moreover, aqueous oxidative stress markers have been shown to correlate with retinal vascular leakage, increased retinal thickness, and early blood–retinal barrier dysfunction, reinforcing their relevance to disease stage and progression [13,40,45,78,84].

#### 3.2.2. Vitreous Biomarkers and Retinal Oxidative Damage

Consistent with findings in aqueous humor, increased concentrations of lipid peroxidation products, oxidized proteins, and DNA damage markers have been identified in vitreous samples from patients with advanced DR.

Elevated vitreous levels of 8-OHdG, MDA, nitrite, and nitrotyrosine indicate severe oxidative and nitrosative stress directly affecting retinal cells. These markers reflect excessive peroxynitrite formation and are closely associated with endothelial dysfunction and impaired NO bioavailability [1,3,4,13,16,24,27].

Concomitantly, vitreous TAC has been shown to be significantly reduced in patients with DR compared with non-diabetic subjects. These alterations are most pronounced in PDR, where vitreous oxidative biomarkers and depleted antioxidant capacity are associated with neovascular activity, fibrous proliferation, and vitreous hemorrhage. Collectively, these findings suggest that vitreous oxidative stress markers and reduced antioxidant defenses reflect ongoing retinal injury and are closely linked to the pathological processes that characterize late-stage disease [13,37,45,78,79,85,86].

#### 3.2.3. Nitrosative Stress Markers and Advanced Retinal Dysfunction

Markers of nitrosative stress, particularly nitrotyrosine, are significantly increased in advanced stages of DR and are closely associated with endothelial dysfunction and impaired NO bioavailability. Elevated nitrotyrosine levels have been detected in vitreous samples and retinal tissues from patients with proliferative disease, indicating excessive peroxynitrite formation. Nitration of vascular and neuronal proteins contributes to capillary non-perfusion, blood–retinal barrier breakdown, and neurodegeneration [1,3,4,13,16,24,45].

#### 3.2.4. EVs as Biomarkers of Oxidative Stress

EVs, particularly small extracellular vesicles or exosomes, serve as sensitive biomarkers of oxidative stress because their secretion rate, quantity, and specific molecular cargo are directly modulated by the redox state of their parent cells [87]. In addition, they encapsulate a real-time signature of cellular oxidative injury, allowing for potential non-invasive diagnosis and monitoring of ocular diseases through liquid biopsies of blood, tears, or vitreous humor. EVs found in tears, blood, and vitreous humor provide a “molecular window” into the progression of DR, offering a non-invasive method for early detection and monitoring [88]. Elevated levels of specific microRNAs, such as miR-15a in plasma or miR-3976 in serum, were found to correlate with initial retinal damage and the clinical stages of DR. Also, proteomic analysis of vitreous EVs has identified upregulated proteins like Lactate Dehydrogenase A and Apolipoprotein B as potential biomarkers for proliferative DR. Similarly, the presence of Muskelin1 in serum EVs has been considered as a telltale indicator used to distinguish DR patients from those with diabetes but no retinopathy [75,89].

## 4. Biomarkers as Predictors of Progression and Therapeutic Response

### 4.1. Prognostic Value of Oxidative Stress Biomarkers

Longitudinal studies suggest that elevated baseline levels of systemic oxidative stress markers may predict the development and progression of DR. Patients with higher circulating levels of lipid and protein oxidation products, such as MDA and AOPPs, together with reduced systemic antioxidant capacity—reflected by lower TAS/TAC, decreased levels of reduced GSH, and reduced activity of key antioxidant enzymes including SOD and GSH peroxidase—are more likely to develop retinopathy or progress to advanced stages. This prognostic association highlights the potential role of oxidative biomarkers in risk stratification and early intervention strategies [13,24,40,45,90].

### 4.2. Modulation of Biomarkers by Therapeutic Interventions

Interventional studies indicate that improved metabolic control and therapies targeting oxidative stress–related pathways can partially modulate oxidative stress biomarkers, particularly in the early-stage DR. Optimization of glycemic control, blood pressure, and lipid metabolism remains the cornerstone of systemic management and is associated with reductions in circulating lipid peroxidation markers, such as MDA, as well as partial restoration of antioxidant defenses, including superoxide dismutase, GSH peroxidase, and total antioxidant capacity. Beyond systemic metabolic control, experimental and clinical studies provide a biological rationale for therapeutic strategies directly targeting oxidative stress and its upstream mechanisms. Antioxidant supplementation with compounds such as vitamins C and E, alpha-lipoic acid, N-acetylcysteine, taurine, and beta-carotene has consistently demonstrated the ability to reduce oxidative stress markers and attenuate retinal damage in cell culture systems and diabetic animal models, whereas evidence from randomized human clinical trials remains heterogeneous and less conclusive.

Indeed, excessive scavenging of ROS may interfere with redox-sensitive protective pathways, including Nuclear Factor Erythroid 2-Related Factor 2 (Nrf2)-mediated antioxidant responses, partially explaining the limited efficacy observed in some clinical studies [91,92,93,94,95,96,97,98,99,100,101].

More mechanistically targeted approaches have shown greater promise in experimental DR. Inhibition of PARP, a key mediator linking mitochondrial oxidative stress to downstream pathogenic pathways, reduces retinal oxidative–nitrosative stress, inflammation, glial activation, and apoptosis in diabetic animal models; however, translation into human clinical application is still under investigation. Similarly, activation of transketolase using thiamine or its derivative benfotiamine redirects glycolytic intermediates away from hyperglycemia-induced damaging pathways, leading to reduced activation of AGEs, PKC and hexosamine pathways and prevention of early microvascular lesions. Pharmacological inhibition of redox-sensitive inflammatory signaling, particularly NF-κB, further attenuates oxidative stress-driven inflammation and neovascular responses in experimental models. Importantly, drugs with pleiotropic metabolic and antioxidant effects have demonstrated clinically meaningful benefits. Fenofibrate, beyond its lipid-lowering action, exerts protective effects on oxidative stress, inflammation, endothelial dysfunction, and neurodegeneration, and human clinical trial data support its role in reducing the progression of DR. Similarly, angiotensin II receptor blockers appear to confer retinal protection in both experimental studies and selected clinical trials, potentially through modulation of oxidative stress, AGEs accumulation, and VEGF signaling.

Despite these advances, in advanced DR, oxidative stress biomarkers often remain elevated despite treatment, suggesting limited reversibility once extensive microvascular and neuroretinal damage has occurred. Collectively, these findings indicate that therapeutic modulation of oxidative stress is most effective when implemented early in the disease course and that future strategies should prioritize targeted, mechanism-based interventions aimed at upstream drivers of redox imbalance rather than nonspecific antioxidant supplementation alone [1,13,45,65,91,92,93,94,95,96,97,98,99,100,101,102].

## 5. Oxidative Stress Biomarkers and Diabetic Macular Edema

Human clinical studies indicate that systemic oxidative stress is increased in patients with DME and tends to parallel the severity of macular involvement. Elevated circulating levels of lipid peroxidation products, such as MDA, as well as markers of protein oxidation, have been reported more frequently in patients with DME compared with diabetic individuals without macular edema. In parallel, reduced systemic antioxidant defenses—including decreased levels of reduced GSH and TAC—have been described in patients with DME, supporting a role for redox imbalance in the pathophysiology of macular fluid accumulation. These alterations are mechanistically linked to endothelial dysfunction and breakdown of the BRB, key processes underlying vascular leakage in DME.

More direct evidence is provided by analyses of ocular fluids. Increased levels of oxidative stress markers, including MDA, protein carbonyls, and 8-hydroxy-2′-deoxyguanosine, have been detected in the aqueous and vitreous humor of patients with diabetic macular edema. These biomarkers have been shown to associate with structural features of macular edema, such as increased central retinal thickness and cystoid changes on optical coherence tomography, and to coexist with elevated inflammatory and angiogenic mediators. Together, these findings support a contributory role of oxidative stress in sustaining vascular leakage and chronic inflammatory signaling within the macula. Emerging evidence further suggests that oxidative stress burden may modulate treatment response in diabetic macular edema. Higher levels of systemic and ocular oxidative stress markers have been observed in patients with suboptimal anatomical or functional response to anti-VEGF therapy, suggesting that persistent oxidative and inflammatory injury may limit the efficacy of anti-angiogenic treatment alone. Although prospective validation is still limited, these observations support the potential utility of oxidative stress biomarkers as adjunctive indicators of disease activity and therapeutic response in DME [3,4,5,14,38,45,103].

## 6. Oxidative Stress Biomarkers and Retinal Ischemia

Retinal ischemia, characterized by capillary non-perfusion and hypoxia, is closely linked to oxidative stress in DR. Oxidative damage to endothelial cells and pericytes promotes capillary destabilization, impaired vascular autoregulation, and progressive capillary dropout, thereby contributing to retinal ischemia. Increased levels of nitrotyrosine, a marker of nitrosative stress and peroxynitrite-mediated protein modification, have been reported in vitreous samples from patients with advanced DR and in experimental ischemic models, supporting a role for nitrosative stress in ischemia-associated vascular injury [41,42,48,70,77,85,104].

In parallel, elevated levels of other oxidative stress biomarkers, including MDA, protein carbonyls, and markers of oxidative DNA damage such as 8-hydroxy-2′-deoxyguanosine, have been detected in the aqueous and vitreous humor of patients with PDR. These findings are complemented by clinical evidence showing reduced levels of key ocular antioxidants in eyes with ischemic retinal disease: decreased vitreous concentrations of vitamin C, a major antioxidant in the intraocular environment, have been associated with greater macular ischemia as assessed by fluorescein angiography, providing indirect clinical support for a link between impaired redox buffering capacity and ischemic retinal damage. Clinically, retinal ischemia has been primarily assessed using fluorescein angiography, particularly ultra-widefield imaging, which enables visualization and quantification of peripheral capillary non-perfusion and calculation of ischemic indices. Although oxidative stress biomarkers have not been directly correlated with angiographically quantified global ischemic burden, their increased expression in advanced ischemia-prone disease stages supports the concept that local redox imbalance accompanies and potentially amplifies microvascular degeneration [77,78,79,84,85,105].

Moreover, reactive oxygen and nitrogen species stabilize hypoxia-inducible factor-1α and promote the expression of angiogenic and inflammatory mediators, including VEGF, which further disrupt vascular integrity and exacerbate ischemia. In this context, oxidative stress acts not only as a marker of disease severity but also as an active participant in the cycle linking hypoxia, microvascular damage, and progressive retinal ischemia [4,13,31,39,45,105].

## 7. Oxidative Stress Biomarkers and Visual Outcomes in Diabetic Retinopathy

Clinical evidence indicates that oxidative stress biomarkers increase with DR severity and are elevated in advanced stages that are typically associated with worse visual outcomes. Cross-sectional clinical studies have shown that systemic markers of lipid peroxidation and nitrosative stress—such as MDA and NO—as well as reduced antioxidant defenses, including decreased levels of reduced GSH, are significantly higher in patients with non-proliferative and proliferative DR compared with diabetic patients without retinopathy. Importantly, in a prospective clinical study stratifying patients according to ETDRS classification, increased plasma levels of nitrosative and oxidative stress markers were directly correlated with reduced best-corrected visual acuity (BCVA), supporting a link between systemic redox imbalance and functional visual impairment, particularly in advanced disease stages.

Overall, these findings indicate that oxidative and nitrosative stress are closely associated with disease severity and functional decline in DR. While most studies support this relationship indirectly through associations with retinopathy stage, selected clinical investigations provide direct evidence that increased oxidative stress burden parallels worsening visual acuity, likely reflecting cumulative damage to the retinal microvasculature and neurosensory retina [17,37,77,84,85,105].

Beyond functional measures, oxidative and nitrosative stress biomarkers have also been linked to in vivo structural retinal alterations detectable by optical coherence tomography. Increased systemic levels of NO and lipid peroxidation products have been shown to correlate with disruption of the photoreceptor inner segment ellipsoid (ISel) band and topographic alterations of the retinal pigment epithelium on spectral-domain OCT imaging. These microstructural changes were more pronounced with increasing severity of DR and were strongly associated with reduced BCVA. Notably, photoreceptor disruption and RPE alterations demonstrated a stronger correlation with visual acuity loss than retinopathy stage alone, indicating that oxidative stress-related neuroretinal damage represents a key structural substrate of visual dysfunction in DR. Collectively, these observations support a mechanistic link between systemic oxidative stress, outer retinal structural integrity, and functional visual outcomes [77].

Emerging evidence supports a critical role of oxidative stress in the neurodegenerative component of DR, which may precede and contribute to visual impairment independently of overt vascular pathology. Experimental and clinical studies indicate that oxidative stress and mitochondrial dysfunction promote early neuronal injury and increased retinal ganglion cell vulnerability, even before classical microvascular lesions become clinically apparent. Structural imaging studies using optical coherence tomography have demonstrated thinning of inner retinal layers and retinal ganglion cell dysfunction in early and intermediate stages of DR, and these neurostructural changes have been associated with reduced best-corrected visual acuity. Although direct correlations between oxidative stress biomarkers and neurodegenerative retinal changes remain limited, the convergence of biochemical, structural, and functional evidence supports a mechanistic link whereby oxidative stress-driven neuronal injury contributes to visual dysfunction alongside, and in part independent of, microvascular damage [2,17,24,26,37].

Taken together, available evidence supports a stage-dependent relationship between oxidative stress biomarkers, retinal structural alterations, and clinical outcomes in DR. Systemic biomarkers primarily reflect global oxidative burden and progression risk, whereas ocular biomarkers provide more direct insight into localized retinal pathology and disease severity. Integrating oxidative stress biomarkers with clinical staging and functional parameters such as BCVA may enhance early diagnosis, improve prognostic accuracy, and support the development of personalized therapeutic strategies. Despite the growing body of evidence, the clinical application of oxidative stress biomarkers remains limited by biological variability, lack of standardization, and partial overlap with other diabetic complications. While systemic markers may capture global metabolic dysregulation rather than retina-specific injury, ocular biomarkers offer greater specificity at the expense of invasive sampling. Nevertheless, combined panels of systemic and ocular oxidative stress biomarkers hold promise for improved risk stratification, disease monitoring, and use as surrogate endpoints in interventional trials targeting redox-related pathways in DR [4,13,16,24,45].

Table 2 summarizes the principal associations between oxidative stress biomarkers and clinical aspects of DR, reporting sample type and corresponding references.

## 8. Sensitivity, Specificity, and Confounding Factors of Systemic Oxidative Stress Biomarkers

Although systemic oxidative stress biomarkers such as MDA, AOPPs, and 8-OHdG consistently correlate with the presence and severity of diabetic retinopathy, their retina specificity remains limited. These markers reflect global redox imbalance and are also elevated in other diabetes-related complications, including diabetic nephropathy, neuropathy, and cardiovascular disease [30,62,106,107,108,109].

Increased circulating levels of systemic oxidative stress biomarkers have been extensively documented in patients with diabetic kidney disease and macrovascular complications, often independently of retinopathy status [30,62,106,107,108,109]. Consequently, systemic oxidative stress markers may capture overall metabolic dysregulation rather than retina-specific injury.

This lack of tissue specificity reduces their diagnostic precision for DR when used in isolation and introduces important confounding factors, including duration of diabetes, renal function impairment, systemic inflammation and concurrent micro- or macrovascular complications.

From a diagnostic sensitivity–specificity perspective, systemic biomarkers may demonstrate good sensitivity for identifying patients at increased risk of microvascular damage but relatively lower specificity for discriminating retinal involvement from other diabetic complications.

In contrast, ocular biomarkers measured in aqueous or vitreous humor provide greater retina-specific insight but are limited by invasive sampling and limited feasibility for routine screening.

These considerations suggest that the clinical utility of oxidative stress biomarkers may be optimized through multimodal strategies integrating systemic markers, ocular biomarkers, structural imaging parameters (e.g., OCT-based neuroretinal alterations), and established clinical staging systems.

## 9. Methodological Limitations of Oxidative Stress Biomarker Studies

Interpretation of oxidative stress biomarkers in diabetic retinopathy is influenced by several methodological limitations that affect reproducibility and clinical translation.

First, variability in biological sampling represents a major source of heterogeneity. Systemic biomarkers are typically measured in serum or plasma, whereas ocular biomarkers are assessed in aqueous or vitreous humor. Differences in compartmental dynamics, blood–retinal barrier integrity, and systemic metabolic influences may significantly affect biomarker levels. In addition, pre-analytical variables—including sample handling, storage conditions, and freeze–thaw cycles—can alter concentrations of markers such as MDA and 8-OHdG [110,111].

Second, oxidative stress assays lack methodological standardization. Biomarkers are quantified using heterogeneous analytical platforms—including spectrophotometric assays, ELISA-based methods, high-performance liquid chromatography, and mass spectrometry—each characterized by different analytical sensitivity and specificity. Inter-assay variability and inconsistent reporting units limit cross-study comparability [112].

Third, substantial heterogeneity in patient populations—including diabetes type, disease duration, glycemic variability, renal impairment, cardiovascular comorbidities, and pharmacological treatment—may independently influence oxidative stress levels. Systemic oxidative markers often reflect global metabolic burden rather than organ-specific injury [62,106].

Finally, most available studies are cross-sectional and rely on single time-point measurements, limiting the ability to distinguish dynamic disease activity from cumulative oxidative damage. Longitudinal validation remains essential before oxidative stress biomarkers can be reliably implemented in routine clinical practice [6].

Together, these methodological considerations underscore the need for standardized sampling protocols, harmonized analytical methodologies, and well-designed prospective studies to define the true clinical utility of oxidative stress biomarkers in diabetic retinopathy.

## 10. Therapeutic Implications and Future Directions

Advances in metabolomics and redox proteomics are enabling the identification of novel oxidative stress-related biomarkers with improved specificity and sensitivity. Integration of oxidative biomarkers with inflammatory, angiogenic, and neurodegenerative markers may provide a more comprehensive molecular signature of DR. Such multidimensional approaches could enhance early detection, guide personalized therapy, and improve understanding of disease heterogeneity.

Given its central pathogenic role, oxidative stress represents an attractive therapeutic target in DR. Antioxidant strategies, including agents targeting mitochondrial dysfunction, NOX inhibition, and enhancement of endogenous antioxidant pathways such as Nrf2 signaling, have shown promising results in preclinical experimental models, although robust efficacy in large-scale human trials has not yet been consistently demonstrated [1,13,113].

In this context, EVs may represent promising candidates and be used not only as biomarkers but also as revolutionary therapeutic tools due to their biocompatibility, low immunogenicity, and unique ability to penetrate biological barriers like BRB. Unlike traditional cell-based therapies, these nanoscale messengers provide a “cell-free” approach that delivers targeted proteins, lipids, and nucleic acids directly to damaged ocular tissues [114].

However, clinical translation remains challenging, likely due to the complexity and compartmentalization of redox signaling. Future therapeutic approaches may require combination strategies that simultaneously target oxidative stress, inflammation, and metabolic dysregulation [1,3,4,13,16,24,45].

Among the oxidative stress biomarkers discussed, some appear more promising for near-term clinical translation. Systemic markers of lipid peroxidation, particularly MDA, together with oxidative DNA damage markers such as 8-OHdG, have shown consistent associations with disease severity and progression across multiple clinical studies. In parallel, measures of TAC may provide complementary information regarding systemic redox reserve.

At the ocular level, aqueous and vitreous concentrations of MDA, 8-OHdG, nitrotyrosine, and reduced antioxidant capacity demonstrate greater retina-specific relevance, particularly in advanced disease and diabetic macular edema. Although invasive sampling limits their routine applicability, these biomarkers may serve as valuable translational endpoints in interventional trials.

Extracellular vesicle-associated microRNAs and proteomic signatures represent an emerging frontier with high specificity potential; however, their standardization and validation remain at an earlier stage compared with classical oxidative stress markers.

## 11. Conclusions

Oxidative stress is a fundamental driver of DR, integrating metabolic, inflammatory, vascular, and neurodegenerative processes. Multiple sources of ROS and impaired antioxidant defenses contribute to sustained retinal injury and disease progression.

Current evidence supports a stage-dependent relationship between oxidative stress biomarkers and clinical outcomes, with systemic markers reflecting global metabolic burden and ocular biomarkers providing greater retina-specific insight. Among available candidates, lipid peroxidation products (particularly MDA), oxidative DNA damage markers such as 8-OHdG, and measures of total antioxidant capacity currently show the strongest clinical consistency and translational potential.

However, limited tissue specificity, biological variability, and lack of assay standardization continue to restrict routine clinical implementation. Future strategies should prioritize multimodal biomarker panels integrating systemic and ocular oxidative markers with structural imaging and functional parameters.

Targeting oxidative stress within a broader, multi-pathway therapeutic framework may represent a key strategy for preventing or slowing the progression of DR.

## Figures and Tables

**Table 1:** Systemic and Ocular Biomarkers of Oxidative Stress in Diabetic Retinopathy.

	Systemic Biomarkers	Ocular Biomarkers (Vitreous/Aqueous Humor)
Markers of Lipid Peroxidation [40,45,78,79,80,81,82]	Malondialdehyde (MDA)	Malondialdehyde (MDA)
	4-Hydroxynonenal (4-HNE)	
Markers of Protein Oxidation [40,45,80]	Advanced oxidation protein products (AOPPs)	Protein carbonyls
	Protein carbonyls	
Markers of oxidative DNA damage [3,45,80,83]	8-Hydroxy-2′-deoxyguanosine (8-OHdG)	8-Hydroxy-2′-deoxyguanosine (8-OHdG)
Antioxidant defense markers [45,78,79,82,84]	Superoxide dismutase (SOD) activity	Vitamin C (ascorbic acid)
	Catalase activity	
	Glutathione peroxidase (GPx) activity	Total antioxidant capacity (TAC)
	Reduced glutathione (GSH)	
	Total antioxidant status/Total antioxidant capacity (TAS/TAC)	
Markers of nitrosative stress/reactive nitrogen species [1,3,4,13,16,24,45]	-	Nitrotyrosine
		Nitric oxide (NO)/nitrite

**Table 2:** Oxidative stress-related biomarkers and associated clinical correlates in diabetic retinopathy.

Biomarker	Associated Clinical Correlate			
	Disease Stage	Retinal Ischemia	Macular Edema	BCVA
Malondialdehyde (MDA)	Correlation (All samples) [62]	Indirect correlation (A and V) [81]	Correlation (All samples) [82]	Correlation (S) [81]
8-Hydroxy-2′-deoxyguanosine (8-OHdG)	Correlation (All samples) [83]	Indirect correlation (A and V) [6]	Correlation (A and V) [83]	No evidence
Advanced oxidation protein products (AOPPs) and Protein carbonyls	Correlation (S and A) [90]	Indirect correlation (A and V) [79]	Correlation (A and V) [79]	No evidence
Nitrotyrosine	Correlation (V) [90]	Indirect correlation (V) [86]	No evidence	No evidence
Nitric oxide (NO)/nitrite	Correlation (A and V) [77]	No evidence	No evidence	Correlation (S) [77]
Total antioxidant capacity (TAC/TAS)	Correlation (S and A) [82]	No evidence	Correlation (S) [78]	No evidence
Vitamin C	No evidence	Correlation (V) [105]	No evidence	No evidence

Abbreviations: S, systemic samples; A, aqueous humor; V, vitreous humor; All samples, biomarker reported in both systemic and ocular compartments.

## Data Availability

The original contributions presented in this study are included in the article. Further inquiries can be directed to the corresponding author.

## Abbreviations

The following abbreviations are used in this manuscript:

4-HNE	4-Hydroxynonenal
8-OHdG	8-Hydroxy-2′-Deoxyguanosine
AGEs	Advanced Glycation End Products
AOPPs	Advanced Oxidation Protein Products
ARB	Angiotensin II receptor blocker
ATP	Adenosine triphosphate
BCVA	Best-Corrected Visual Acuity
BRB	Blood–Retinal Barrier
DAG	Diacylglycerol
DHA	Docosahexaenoic Acid
DME	Diabetic macular edema
DR	Diabetic Retinopathy
ETDRS	Early Treatment Diabetic Retinopathy Study
EVs	Extracellular vesicles
GAPDH	Glyceraldehyde-3-Phosphate Dehydrogenase
GSH	Reduced Glutathione
HIF-1α	Hypoxia-Inducible Factor 1 Alpha
MDA	Malondialdehyde
MMPs	Matrix Metalloproteinases
mtDNA	Mitochondrial DNA
NAD^+^	Nicotinamide adenine dinucleotide
NADH	Reduced nicotinamide adenine dinucleotide
NADPH	Nicotinamide Adenine Dinucleotide Phosphate (Reduced Form)
NF-κB	Nuclear Factor Kappa-Light-Chain-Enhancer of Activated B Cells
NO	Nitric oxide
NOX	NADPH Oxidase
NPDR	Non-Proliferative Diabetic Retinopathy
NRF2	Nuclear Factor Erythroid 2–Related Factor 2
OCT	Optical Coherence Tomography
OXPHOS	Oxidative Phosphorylation
PARP	Poly(ADP-ribose) Polymerase
PDR	Proliferative Diabetic Retinopathy
PKC	Protein Kinase C
PUFAs	Polyunsaturated Fatty Acids
RAGE	Receptor for Advanced Glycation End Products
RNS	Reactive Nitrogen species
ROS	Reactive Oxygen Species
RPE	Retinal pigment epithelium
SD-OCT	Spectral-domain optical coherence tomography
SOD	Superoxide Dismutase
TAC	Total antioxidant capacity
TAS	Total antioxidant status
VEGF	Vascular Endothelial Growth Factor
